# Supplementary material for: A high-resolution mRNA expression time course of embryonic development in zebrafish
Source: eLife. 2017 Nov 16;6:e30860. doi: 10.7554/eLife.30860 (PMC5690287; doi:10.7554/eLife.30860)
Supplement: Supplementary file 6. [file elife-30860-supp6.zip › biolayout-clusters-files/Cluster143-genes.html]

Cluster143


# Cluster143: Genes

| Ensembl ID | Gene Name | Chr | Start | End | Biotype |
| --- | --- | --- | --- | --- | --- |
| ENSDARG00000099425 | CFI | KN150402.1 | 17609 | 42424 | protein\_coding |
| ENSDARG00000042780 | apoba | 17 | 30687105 | 30711003 | protein\_coding |
| ENSDARG00000022767 | apobb.1 | 20 | 31366899 | 31382011 | protein\_coding |
| ENSDARG00000012694 | c3a.1 | 1 | 55460926 | 55507680 | protein\_coding |
| ENSDARG00000021004 | c5 | 5 | 62764794 | 62816203 | protein\_coding |
| ENSDARG00000025703 | dmgdh | 21 | 97366 | 127688 | protein\_coding |
| ENSDARG00000024160 | hao1 | 17 | 3879456 | 3912544 | protein\_coding |
| ENSDARG00000091465 | si:dkey-66a8.7 | 6 | 37473447 | 37482284 | protein\_coding |
| ENSDARG00000070168 | sod3a | 7 | 71096760 | 71105633 | protein\_coding |
